# Is There Agreement Between Clinical Outcomes as Perceived by the Surgeon and the Patient in Revision Total Hip Arthroplasty?

**DOI:** 10.3390/jcm14217488

**Published:** 2025-10-22

**Authors:** Víctor Casas-Gallego, Miguel A. Ortega, Basilio J. de la Torre-Escuredo

**Affiliations:** 1 Service of Traumatology of University Hospital Ramón y Cajal, 28034 Madrid, Spain; vcasasgallego@gmail.com (V.C.-G.); bjtorre@gmail.com (B.J.d.l.T.-E.); 2 Ramón y Cajal Institute of Sanitary Research (IRYCIS), 28034 Madrid, Spain; 3 Departments of Medicine and Medical Specialities, Faculty of Medicine and Health Sciences (CIBER-EHD), University of Alcalá, Alcalá de Henares, 28805 Madrid, Spain; 4 Department of Surgery, Medical and Social Sciences, Faculty of Medicine and Health Sciences, University of Alcalá, Alcalá de Henares, 28805 Madrid, Spain

**Keywords:** hip, arthroplasty, surgeon’s evaluation

## Abstract

**Objectives**: Revision total hip arthroplasty (rTHA) is a complex surgery with variable functional outcomes that often differ between the surgeon’s perception and the patient’s experience. Therefore, the aim of this study is, first, to evaluate functional outcomes based on the reason for revision, type of revision, acetabular defect, and number of prior revision surgeries; and second, to compare outcomes from both the surgeon’s and the patient’s perspectives to determine whether or not there is agreement between them. **Materials and Methods**: An observational study was conducted on patients who underwent rTHA at a tertiary-level center from January 2013 to December 2018, with a median follow-up of 41 months. A total of 149 procedures were performed during this period. The variables analyzed included the indication for revision surgery, type of revision, presence of acetabular defect, and number of previous revision surgeries. The surgeon’s perspective was assessed using the Harris Hip Score (HHS), while the patient’s perspective was evaluated using the Western Ontario and McMaster Universities Osteoarthritis Index (WOMAC) and the Short Form-12 Health Survey (SF-12). **Results**: Analysis of the variables from both surgeon’s and patient’s perspectives showed statistically significant differences regarding the indication for revision and the SF-12 component, with patients undergoing revision for infection or dislocation reporting worse functional outcomes. Although the remaining variables did not reach statistical significance, the surgeon perceived worse outcomes in patients revised for infection and in those who underwent revision of both components (acetabular and femoral). Conversely, patients reported poorer functional outcomes when operated on for infection or dislocation, when both components were revised, and when they had undergone more than one revision surgery. Additionally, a statistically significant trend was observed showing worse outcomes with increasing anesthetic risk. Linear regression analysis between the surgeon’s evaluation and the patient-reported outcome measures showed a statistically significant association, indicating that higher surgeon scores correlated with fewer symptoms and better hip function as reported by patients. **Conclusion**: There was concordance between the surgeon’s evaluation, measured by the Harris Hip Score (HHS), and the patient’s perception of health status through PROMs, specifically the SF-12 and WOMAC questionnaires. Although overall results were satisfactory regardless of the reason for the revision, type of revision, defect grade, or number of revisions; outcomes were slightly worse in patients revised for dislocation or infection, those undergoing revisions of both components, and in cases involving multiple revision surgeries.

## 1. Introduction

Revision total hip arthroplasty (rTHA) has become a routine procedure in orthopedic surgery departments due to the high number of primary total hip arthroplasties (THAs) performed and the notable re-revision rate, which ranges between 7% and 17% depending on the series [1,2].

Total hip arthroplasty itself is one of the most frequently performed procedures in orthopedic surgery, with an incidence of 106 cases per 100,000 inhabitants in Spain [2]. The incidence of primary procedures is about 1.5 times higher in women [3], and nearly 65% of patients undergoing total hip replacement are over 65 years of age [3]. As the number of primary procedures continues to rise, together with increasing life expectancy, the demand for rTHA is expected to grow steadily in the coming years.

These procedures are typically performed in elderly patients with compromised overall health. Moreover, they are technically demanding surgeries that may involve significant blood loss. Thus, they represent a surgical challenge with inconsistent functional outcomes.

The most common indications for revision surgery include loosening of the prosthetic components—either septic or aseptic—and prosthetic instability [4,5,6]. The fundamental goals of this surgery are based on three key principles: restoring an appropriate center of rotation (CoR) [7], achieving stable fixation of the implants to the remaining bone, whether cemented [8] or non-cemented [9]; and, when possible, restoring lost bone stock [4]

The scientific literature regarding rTHA outcomes lacks comprehensive data on the relationship between patient-reported outcome measures (PROMs) and different surgical factors. In general, studies published to date consist of national registry series or case series that evaluate mid- to long-term outcomes without examining their relationship to the type of revision, reason for revision, or center of rotation achieved during reconstruction [5].

In some cases, radiographic outcomes may appear satisfactory, but the patient does not always report a favorable functional result. This highlights the importance of developing tools to assess patients’ quality of life, which can help inform risk-benefit discussions, refine clinical indications, and identify patients who may not be ideal candidates for such procedures [10].

Therefore, given the lack of consensus in the literature regarding clinical and radiological outcomes in rTHA, we conducted a retrospective cohort study of patients who underwent total or partial revision (acetabular and/or femoral) of prosthetic components at a tertiary care center. We hypothesized that the functional outcome perceived by the patient following rTHA may not align with the clinical-radiological outcomes interpreted by the surgeon. Additionally, we examined whether surgical variables such as reason for revision, type of revision (acetabular, femoral, or both components), number of revisions, and acetabular defect significantly influenced functional outcomes. Finally, we also assessed whether the American Society of Anesthesiologists (ASA) physical status classification, which reflects the patient’s health status before surgery, had an impact on the outcomes.

## 2. Materials and Methods

### 2.1. Study Design

An analytical observational study was carried out with data from patients who underwent rTHA. The study was approved by the Research Ethics Committee (CEIM registration number: 282/20). All patients were informed about the purpose of the study and provided informed consent for participation.

### 2.2. Patients

Patients who underwent rTHA between January 2013 and December 2018 were included. A total of 251 procedures in 243 patients were carried out. Patients included in the study were 149. An acetabular component revision was performed in 73 patients, a femoral component revision in 32 patients, and both components were revised in 44 patients. Cases were collected during 2021, and the median follow-up was 41 months.

The exclusion criteria were age below 18 years; cases involving revision of mobile components; surgery primarily due to cancer; osteosynthesis failure in the femur; and refusal to participate in the study.

The data collected from the patients included age, sex, body mass index (BMI), diabetes mellitus status, anesthetic risk according to the American Society of Anesthesiologists (ASA) scale, reason for revision surgery (septic-aseptic loosening, instability), type of revision (acetabular, femoral, or both), number of revision surgery previously; and type of acetabular defect. Similarly, complications such as periprothetic infection, dislocation, and mortality were analyzed. All the data were collected by one of the authors during the clinical interview.

### 2.3. Assessment of Bone Defects

To assess acetabular defects, these were classified into mild and severe defects according to the Saleh and Paprosky classifications [10,11]. According to Paprosky’s classification, mild defects are those defects whose reconstruction involve an intact acetabular tear drop (Paprosky type I to IIB), and severe defects include those with involvement of the tear drop, acetabular fundus, and posterolateral region (Paprosky IIC to IIIB). According to Saleh, mild defects include contained defects presenting with an intact acetabular fundus and columns (Saleh I–II), and severe defects include uncontained acetabular defects (Saleh III–V).

### 2.4. Evaluation of Functional Outcomes

Three questionnaires were administered. From the orthopedic surgeon’s perspective, the Harris Hip Score (HHS) [12,13] was collected, and the patient’s overall satisfaction with the procedure was inquired about. To assess patient-reported outcomes, the Western Ontario and McMaster Universities Osteoarthritis Index (WOMAC) [14] functional quality of the hip questionnaire and the Short Form 12 (SF-12) quality of life questionnaire, with its two components -physical and mental- [15], were provided to be completed by the patients with the assistance of their companions.

### 2.5. Statistical Analysis

Statistical analysis was performed using STATA/MP version 17.0 (College Station, TX, USA). Results are presented as mean, median, and standard deviation. The Chi-square test and Fisher’s exact test were used for nominal variables. For the analysis of independent variables with parametric distribution, Student’s *t*-test and one-way ANOVA were used, with Bonferroni post hoc tests for multiple group comparisons. For multiple evaluations, multiple linear regression analysis was performed. A *p*-value < 0.05 was considered statistically significant.

## 3. Results

### 3.1. Patient Demographics

Table 1 presents the descriptive variables of the 149 patients included in our sample.

### 3.2. Analytical Results

#### 3.2.1. Surgeon-Reported Outcomes

##### Implant Survival

**Figure 1:**
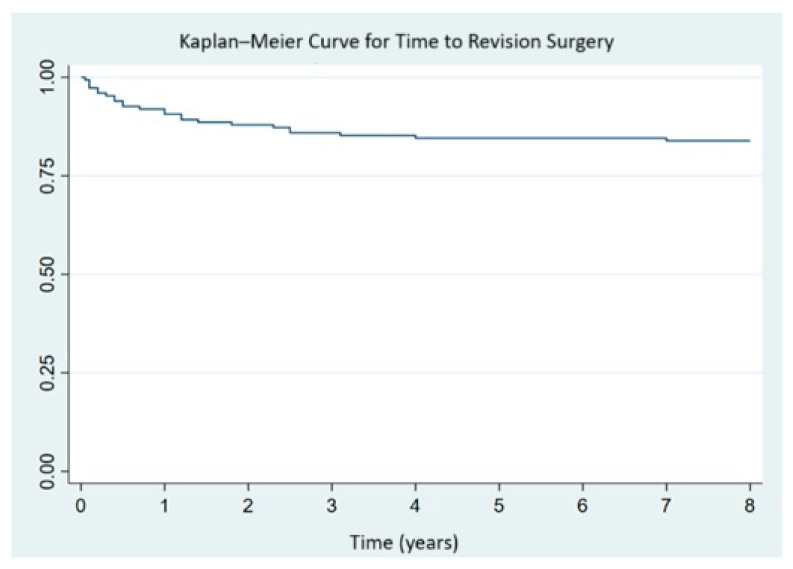
Kaplan–Meier survival curve of the implants.

Implant survival was evaluated in 148 patients, as one patient suffered an intraoperative complication resulting in death. The survival curve shows an 84.46% implant survival rate at 3.41 years (Figure 1).

The most frequent complications requiring reoperation were prosthetic dislocation (8 patients) and infection (8 patients).

### 3.3. Functional Results (HHS)

**Figure 2:**
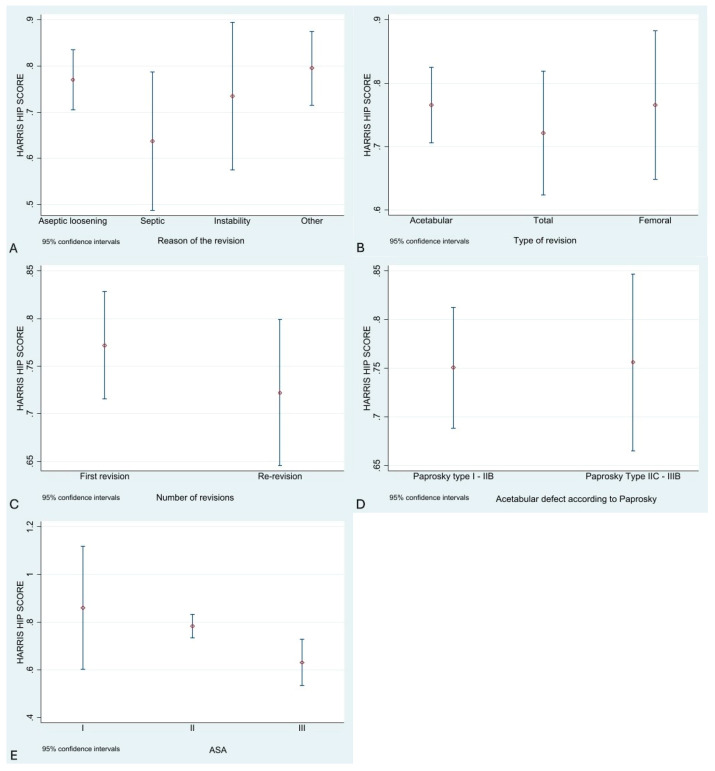
Boxplots were generated for each variable studied and functional outcome (HHS). Graph (**A**): Revision indication. Graph (**B**): Revision type (acetabular, femoral, or total). Graph (**C**): Number of prior revisions. Graph (**D**): Acetabular defect. Graph (**E**): ASA anesthetic risk scale. Each graph displays the group estimate and its confidence interval.

To compare groups by revision indication, cases were classified into four categories: aseptic loosening, periprosthetic infection, instability, and others. A one-way ANOVA was performed, showing no statistically significant differences between groups (F = 1.82, *p* = 0.15). Bonferroni post hoc tests also showed no significant differences. However, there were clinically relevant differences, with patients previously diagnosed with infection having the worst outcomes (Figure 2A).

Regarding the type of revision—acetabular, femoral, or both—no statistically significant differences were found (F = 0.36, *p* = 0.70), though patients undergoing total component revision had worse functional outcomes (Figure 2B).

To evaluate functional outcomes in relation to the number of previous revisions, patients were divided into two groups: those with no prior revision surgery and those with at least one previous revision. Patients undergoing two-stage infection revision were classified in the latter group. No statistically significant differences were found (t = 1.05, *p* = 0.30), though better function was observed in patients without previous revisions (Figure 2C).

Acetabular defect severity, assessed using the Paprosky classification, showed no significant differences (t = –0.10, *p* = 0.92). The Saleh classification also showed no significant differences (t = 0.34, *p* = 0.73), although patients with more severe defects had worse function (Figure 2D).

Significant differences were found between functional results and anesthetic risk (ASA classification) (F = 5.19, *p* = 0.008), showing poorer outcomes with increasing anesthetic risk (Figure 2E).

### 3.4. Patient-Reported Outcomes

According to revision indication and the SF-12 physical component, statistically significant differences were found (F = 2.70, *p* = 0.05). Patients with a prior diagnosis of infection or dislocation had worse functional results than those with aseptic loosening or other causes. No significant differences were found in the mental component (F = 0.15, *p* = 0.93).

Using the WOMAC questionnaire, no significant differences were found by revision indication (F = 0.51, *p* = 0.67).

Regarding revision type, there were no significant differences (F = 0.89, *p* = 0.41) but patients undergoing revision of both components had lower average scores than those with a single component revision. No significant differences were found in the mental aspect (F = 0.12, *p* = 0.88), nor in WOMAC scores among acetabular, femoral, and total revisions (F = 0.36, *p* = 0.70).

**Figure 3:**
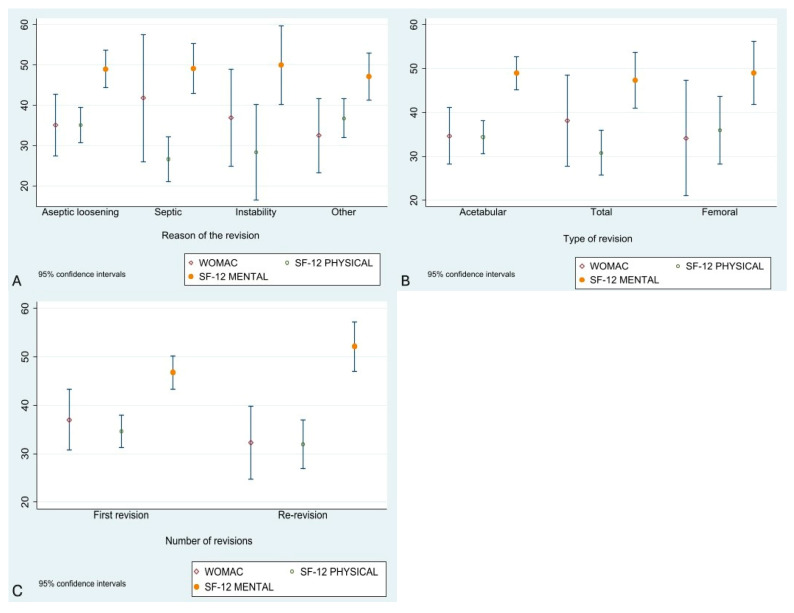
Boxplots were generated for SF-12 physical, SF-12 mental, and WOMAC: Graph (**A**): Revision indication. Graph (**B**): Revision type (acetabular, femoral, or total). Graph (**C**): Number of prior revisions. Each graph shows group estimates and confidence intervals.

When comparing by number of prior revisions, no significant differences were found (T = 0.90, df = 71, *p* = 0.37). However, patients with more than one revision had worse functional outcomes. In the mental component, no significant differences were found (T = –1.80, df = 71, *p* = 0.07), although results approached significance, with better mental scores in patients who had undergone at least one prior revision. WOMAC also showed no significant differences between first revisions and re-revisions (T = 0.91, *p* = 0.36, df = 71) (Figure 3).

A significant relationship was found between the surgeon’s assessment (HHS) and the patients’ perception of health status using PROMs (SF-12 physical and mental components, and WOMAC).

Linear regression analysis between HHS and WOMAC showed a statistically significant association (F = 61.3, *p* < 0.001, adjusted R^2^ = 0.45), indicating that higher surgeon-assessed scores correlated with fewer symptoms and better hip function reported by patients.

**Figure 4:**
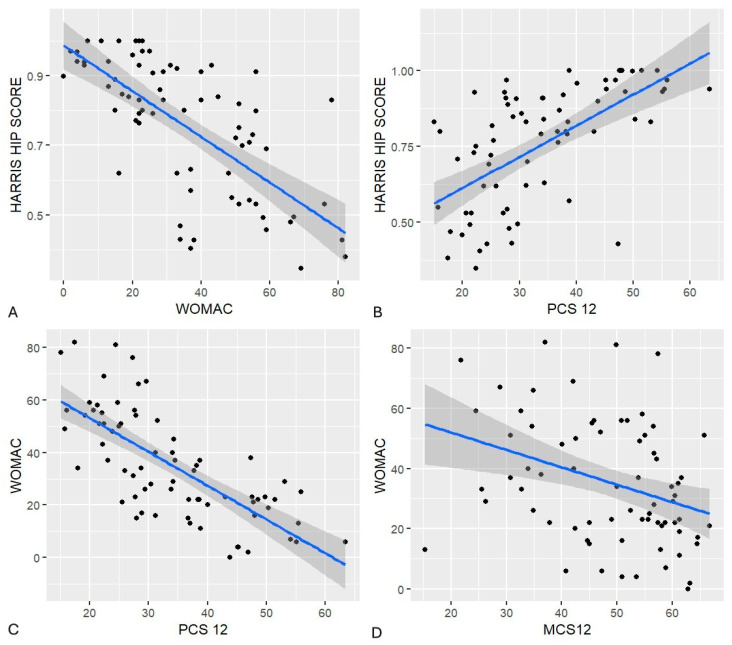
Scatter plots were generated: Graph (**A**): HHS vs. WOMAC. Graph (**B**): HHS vs. SF-12 physical. Graphs (**C**,**D**): WOMAC vs. SF-12 physical and mental. Across all comparisons, greater hip dysfunction correlated with poorer overall function.

Similarly, the significant correlation between PROMs (WOMAC and SF-12) suggests that patients’ perceived limitations in daily activities align with clinical evaluations. These results highlight the importance of combining clinician and patient perspectives to gain a comprehensive understanding of recovery and functional impact (Figure 4).

### 3.5. Complications

#### 3.5.1. Infection

A total of 7 infections (4.7%) were observed. A significant association was found between infection and revision type (*p* = 0.01, Fisher’s test). Patients undergoing femoral revision (tr = 0.10) or total component revision (tr = 0.10) had a higher relative infection rate compared to those with only acetabular revision (tr = 0).

No significant associations were found with revision indication (*p* = 0.11), number of previous revisions (*p* = 0.13), or acetabular defect severity according to Paprosky (*p* = 0.16) or Saleh (*p* = 0.09).

#### 3.5.2. Dislocation

No significant differences were found in implant stability based on revision indication (*p* = 0.20), type of components revised (*p* = 0.42), number of revisions (*p* = 0.17), or acetabular defect severity (Paprosky *p* = 0.87, Saleh *p* = 0.34).

#### 3.5.3. Mortality

Significant associations were found between mortality and patient age (*p* < 0.001), anesthetic risk (ASA scale, *p* < 0.001), as well as occurrence of prosthetic dislocation (*p* = 0.04) and fracture (*p* = 0.008). No significant association was found with post-revision infection (*p* = 0.11).

## 4. Discussion

When facing revision surgeries of total hip arthroplasty, especially in the most demanding cases with severe bone loss, there are often discrepancies between the clinical and radiological outcomes perceived by the surgeon and the functional outcomes perceived by the patient. In other words, despite having a reconstruction that could be considered anatomical—with restoration of the center of rotation and stable implants—patients often report functional outcomes that do not align with radiological findings.

For this reason, there has been longstanding interest in identifying predictors of success in hip revision surgery. Factors such as the reason for revision surgery, age, sex, and the number of previous revisions have been shown to correlate with functional outcomes [16]. Davis et al. were among the first to evaluate this, demonstrating that good preoperative function predicts better postoperative outcomes [17]. Davis et al. were among the first to evaluate this, demonstrating that good preoperative function predicts better postoperative outcomes [18]. Conversely, other studies, such as those by Innocenti and Harada, did not find differences based on the reason for revision after two years of follow-up [4,19].

Our study, after analyzing potential outcome differences from the surgeon’s perspective—based on the reason for revision, the type of revision (acetabular, femoral, or both components), and the number of revision surgeries—did not show clinically significant differences. Nonetheless, although not statistically significant, there was a trend toward better functional results in patients whose reason for revision was aseptic loosening. This may be related to the morbidity associated with two-stage revision surgeries, which were performed in all patients with septic loosening. This reasoning is also highlighted in other studies [5,17,18]. Likewise, we observed worse outcomes in cases where both components were revised [17,20,21].Regarding the number of previous revisions, patients without prior revision surgeries showed better function [17,18,19].

The lack of statistical significance, despite a trend toward it, may be due to limited statistical power. Increasing the number of cases might reveal statistically significant differences.

Regarding bone defects classified according to Paprosky [12], we decided to group them by complexity: severe defects (IIC to IIIB) and mild-to-moderate defects (I to IIB). In our series, no differences were observed in hip-specific function (HHS). These findings are consistent with published literature. Driscoll et al., in a prospective study, argue that patients with greater preoperative bone defects present worse preoperative function and are the ones who perceive the most benefit after surgery. This author also found no relevant differences in final functional outcome and the severity of the acetabular defect [22].

We assessed anesthetic risk using the ASA classification and evaluated clinical function during follow-up with the Harris Hip Score. We did find significant results relating functional outcomes and ASA anesthetic risk. To our knowledge, there are no previous studies in the literature supporting this specifically in the context of revision surgery. However, in primary hip arthroplasty, there are studies that show similar results to ours [23,24].

Patient-reported outcomes (PROMs) have been shown to correlate with preoperative expectations and final outcomes [25]. In this study, the HHS was completed by the clinical researcher, while the WOMAC and SF-12 were self-administered by the patients to avoid observer bias. Notably, we found significant functional differences in patients revised for infection and instability. In the case of infection, the two-stage revision—along with the interim period during which patients ambulate with a spacer—results in decreased functional outcomes [26]. Regarding the mental component, significant differences were also observed based on the number of revisions. Patients undergoing multiple surgeries showed better mental health. This may be because, despite worse motor function, they underwent further revision with the goal of improving overall function.

When comparing overall outcomes from both the surgeon and patient perspectives, there is clear concordance among the questionnaires. That is, better functional outcomes reported by the patient in the WOMAC and SF-12 correspond to higher scores in the HHS as recorded by the clinician. These results are similar to those in patients undergoing primary hip arthroplasty, but to our knowledge, have not been studied in hip revision surgery [27,28]. Furthermore, as seen in the relationship between function and the mental component of the SF-12, lower scores in the physical SF-12 and WOMAC are associated with worse results in the mental SF-12. This suggests that poor physical function may be linked to a greater risk of anxiety or depression—similar to findings in the knee pathology literature [29,30].

The present study provides a novel perspective by directly comparing the surgeon’s clinical assessment with patient-reported outcomes in the context of revision total hip arthroplasty. While previous studies have analyzed functional or radiological results separately, few have explored whether the surgeon’s perception of success aligns with the patient’s subjective experience. This dual evaluation adds clinical value by integrating both viewpoints, offering a more comprehensive understanding of postoperative recovery and satisfaction. From a practical standpoint, the observed concordance between the Harris Hip Score and PROMs such as the WOMAC and SF-12 suggests that the surgeon’s clinical assessment can reliably reflect patient-perceived function, facilitating postoperative follow-up when PROM data are unavailable. Moreover, the identification of specific subgroups—such as patients revised for infection or dislocation, or those with higher ASA risk—as having poorer outcomes underscores the need for tailored perioperative management and more precise preoperative counseling. These findings contribute to refining the prognostic framework for revision hip surgery and highlight the importance of incorporating patient-centered measures into surgical evaluation and decision-making.

In our series, early mortality was 2.7%, a figure higher than that reported in the literature. However, our patients had a higher mean age compared to other studies, which could explain this difference [31,32,33,34]. Mortality risk was significantly higher in ASA III patients (35.5%) compared to ASA II or I. Therefore, as shown in other studies [35,36] the ASA classification is a predictive factor of life expectancy following rTHA. Similarly, complications such as periprosthetic fracture or instability significantly affected mortality. These findings are consistent with other series in which a complication may lead to a fatal outcome [31,35]. Regarding age, we observed a clear association with mortality, which is expected due to higher comorbidities and frailty in older patients. Raddcliffe et al. reported a mortality of 1.7% in patients under 75 years old, compared to 13.3% in those over 75 [37]. Similar findings have been observed in other studies [33,37,38]. For all these reasons, despite patient optimization, rTHA poses higher risks in patients with elevated anesthetic risk and older age, which reinforces the need to optimize these patients preoperatively before undergoing revision surgery [34,39].

Infection risk has been extensively studied to reduce its incidence. In our sample, we could not statistically demonstrate a higher infection risk in patients with prior infection, although these patients did have a higher relative risk compared to others. This is likely due to limited statistical power. Our results are comparable to those of Innocenti et al., who reported a 20% reinfection risk in patients with previous infections [5]. Another notable finding is the significant increase in infection depending on the type of revision, with a higher incidence in patients undergoing total component revision compared to single-component revisions. These results should be interpreted with caution but seem reasonable, as total revisions are longer procedures with greater exposure of the surgical site.

The findings should be interpreted considering several limitations. The preoperative functional status of the patient was not analyzed, as the primary objective of this study was to assess whether, regardless of various factors—patient-related or surgical—these patients could benefit functionally from hip revision surgery. Additionally, we acknowledge the limited number of patients, which may explain why some results, though trending toward significance, were not statistically significant. Patients in this cohort were heterogeneous, with variability in revision indication, type of revision, and defect severity, among others, which could limit the precision of comparisons. Although our follow-up is short, we consider it comparable to other studies on revision surgery [5,18,40].

## 5. Conclusions

In conclusion, the patient’s perception, assessed via PROMs (WOMAC and SF-12), is consistent with the clinician’s perception in patients undergoing hip revision surgery. From a functional standpoint, outcomes are satisfactory regardless of the reason for revision, revision type, defect grade, or number of previous revisions. However, outcomes were slightly worse in patients revised for dislocation or infection, those undergoing revisions of both components, and in cases involving multiple revision surgeries. Furthermore, patients with higher anesthetic risk exhibit poorer functional outcomes and a greater risk of mortality following hip revision surgery. Larger-scale registry studies are needed to draw more robust conclusions.

## Figures and Tables

**Table 1:** Descriptive Analysis of Patient Demographics and Clinical Variables. The results are presented as means ± standard deviations (SDs) for continuous variables, and as frequencies and percentages for categorical variables. The table includes data on age, body mass index (BMI), sex (female), ASA classification, type of revision, reason for primary arthroplasty and revision, acetabular defect classification, and scores from the WOMAC scale, Harris Hip Score, and SF-12 questionnaire. Comparisons were made for age, weight, BMI, WOMAC score, Harris Hip Score, SF-12 score, and the incidence of post-revision complications, including infection, dislocation, and mortality.

	Mean or *n* (SD)	Min–Max or %
**Age**	74.93 (±13.38)	34–97
**BMI**	26.96 (±6.49)	16.6–44
**Type of revision**		
− Acetabular	73	49.0%
− Femur	32	21.47%
− Total	44	29.53%
**Sex**	Male: 66	44.3%
	Female: 83	65.7%
**ASA**	ASA I: 10	6.71%
	ASA II: 91	61.07%
	ASA III: 45	30.2%
	ASA IV: 3	2.01%
**Reason for primary arthroplasty**	Coxarthrosis: 78	55.71%
	NAV: 11	7.85%
	Fracture: 26	18.67%
	Other: 25	16.10%
**Reason for revision**	Dislocation: 18	12.08%
	Fracture: 8	5.37%
	Loosening: 63	42.28%
	Wear: 12	8.05%
	Pain: 8	5.37%
	Infection: 20	13.42%
	Rupture: 8	5.37%
	Cotyloiditis: 3	2.01%
	Other: 9	6.04%
**Acetabular defect according to Paprosky**	I–IIB: 88	75.23%
	IIC–IIIB: 29	24.77%
**Acetabular defect according to Saleh**	I–II: 83	70.94%
	III–V: 34	29.06%
**Functional assessment**	mean (SD)	Min–Max
**Harris Hip Score**	0.75 (0.20)	0.34–1
**WOMAC total**	35.47 (20.72)	0–82
**SF-12**		
− **Physical**	33.72 (11.71)	15.02–63.46
− **Mental**	48.53 (12.20)	15.26–66.67
**Complications**		n (%)
**Post-revision infection**		Early (1 month): 3 (2.04%) Late: 4 (2.72%)
**Hip dislocation**		Early (3 months): 13 (8.78%) Late: 3 (2.04%)
**Mortality**		Early: 4 (2.01%) Total: 29 (19.59%)

## Data Availability

The data supporting the findings of this study are available from the corresponding author upon reasonable request.

## Abbreviations

BMI	Body Mass Index
CoR	Center of Rotation
HHS	Harris Hip Score
RR	Relative Risk
rTHA	Revision Total Hip Arthroplasty
SF-12	Short Form 12
WOMAC	Western Ontario and McMaster Universities Arthritis Index
